# COGNITIVE FUNCTION IN COUPLES AND COLLABORATIVE INVOLVEMENT IN TYPE 1 DIABETES MANAGEMENT

**DOI:** 10.1093/geroni/igz038.967

**Published:** 2019-11-08

**Authors:** Cynthia Berg, Yana Suchy, Nancy Allen, Rob Kent de Grey, MaryJane Campbell, Vicki Helgeson

**Affiliations:** 1 University of Utah, Salt Lake City, Utah, United States; 2 University of Utah, Salt Lake City, United States; 3 Carnegie Mellon University, Pittsburgh, Pennsylvania, United States

## Abstract

Managing type 1 diabetes involves coordinating complex daily behaviors that benefit from higher cognitive function. One’s spouse’s cognitive function may also be beneficial as spouses may collaborate in daily adherence behaviors and may be especially beneficial for older adults who may be experiencing poorer cognitive function. We examined: 1) whether one’s own and one’s spouse’s cognitive function predicted lower (better) HbA1c, 2) whether collaborating with a more cognitively capable spouse was especially beneficial, and 3) whether the benefit of partners’ cognitive ability occurred through better adherence. 199 couples were recruited where one member was diagnosed with type 1 diabetes for at least one year (52% females, average age 46.8 years, range 25.9-74.9, average duration of diabetes 27 years). Both patients and spouses completed the information subtest from the Wechsler Adult Intelligence Scale-Fourth Addition as a measure of general intelligence. Patients rated the collaborative involvement of their spouse in their diabetes and their adherence to their medical regimen. Multiple regressions revealed that spouse’ higher intelligence uniquely and solely predicted better HbA1c over patient’s intelligence. Collaborating with a spouse of lower intelligence was associated with higher HbA1c for older adults; collaborating with a spouse of higher intelligence was associated with somewhat lower HbA1c. Mediational analyses indicated that spouse’s intelligence was associated with higher HbA1c through better adherence behaviors. The results suggest that individuals with type 1 diabetes who have a spouse of lower cognitive function may benefit from support from others in their network to manage their diabetes.

